# Association between triglyceride glucose-body mass index with all-cause and cardiovascular mortality in adults with osteoporosis: a prospective study

**DOI:** 10.3389/fendo.2025.1649964

**Published:** 2025-10-14

**Authors:** Yuhao Li, Xiaowan Xie, Yazhou Liu, Haoran Sun, Zhaoqi Gong, Wenbo Ding

**Affiliations:** ^1^ Department of Orthopedics, Dandong Central Hospital, China Medical University, Dandong, China; ^2^ Department of Oncology, Dandong Central Hospital, China Medical University, Dandong, China; ^3^ Department of Orthopedics, Dandong Central Hospital, Dalian Medical University, Dandong, China

**Keywords:** all-cause mortality, cardiovascular mortality, TyG-BMI index, osteoporosis, insulin resistance

## Abstract

**Purpose:**

This study aims to examine the relationship between the insulin resistance (IR) biomarker, specifically triglyceride-glucose body mass index (TyG-BMI), and all-cause as well as cardiovascular mortality in patients diagnosed with osteoporosis.

**Methods:**

This study employed a prospective cohort design involving 302 patients diagnosed with osteoporosis, recruited between 2018 and 2020, with follow-up extending until 2024. During this period, occurrences of all-cause mortality (64 cases) and cardiovascular mortality (19 cases) were recorded. A comparative analysis was conducted in conjunction with other insulin resistance indices, including TyG, METS-IR, and TG/HDL-C. Restricted cubic spline and multivariable Cox proportional hazards regression analyses were utilized to elucidate the relationship between the TyG-BMI index and the risk of all-cause and cardiovascular mortality in patients with osteoporosis. Furthermore, subgroup analyses were performed to examine potential interactions and identify subpopulations at elevated risk of mortality.

**Results:**

TyG-BMI is significantly positively correlated with all-cause mortality (for each 1 unit increase, HR = 1.01, 95% CI: 1.00-1.02). Patients in the fourth quartile (Q4) have an almost 2.8-fold increased risk of all-cause mortality compared to those in the first quartile (Q1) (HR = 2.79, 95% CI: 1.16-6.73). The cardiovascular mortality rate is significantly higher in the TyG-BMI Q4 group (HR = 6.33, 95% CI: 1.19-33.80). ROC curve and DeLong test indicate that the predictive capacity of TyG-BMI for all-cause and cardiovascular mortality surpasses that of other insulin resistance indicators. Subgroup analyses further suggest that the risk of cardiovascular mortality is elevated in patients with low HDL, high serum calcium, and elevated creatinine levels.

**Conclusion:**

The TyG-BMI index exhibits a linear association with the risk of both all-cause mortality and cardiovascular mortality. Additionally, TyG-BMI functions as an independent predictor of mortality risk in patients with osteoporosis, with elevated values indicating a poorer prognosis. These findings highlight the significant role of IR in the progression of osteoporosis.

## Introduction

Osteoporosis is a systemic disease characterized by osteopenia and fragility fractures (1–3). Due to its clinically asymptomatic nature, high incidence, and chronic progression, it is often referred to as the “silent epidemic of the 21st century” (4, 5). With an aging population, the global prevalence of osteoporosis continues to rise (6). Osteoporosis results in bone loss, significantly increasing the risk of fractures (1, 3, 7, 8), which not only imposes a substantial burden on healthcare systems but also markedly diminishes the quality of life for affected individuals (9). The incidence of osteoporotic fractures, re-fracture rates, overall mortality, and cardiovascular mortality are notably high, with the lifetime fracture risk reaching up to 50% (7, 10–12). Therefore, the identification of prognostic biomarkers for osteoporosis to predict and mitigate the risk of long-term mortality, particularly cardiovascular-related mortality, remains a critical area of research.

The primary manifestations of metabolic syndrome (MetS) include metabolic dysfunction and central obesity, with the pathophysiology primarily attributed to insulin resistance (IR) (13). Research has demonstrated that MetS is increasingly recognized as an emerging risk factor for the reduction of bone mineral density (BMD) (14). IR arises from the disruption of multiple molecular pathways, leading to decreased insulin sensitivity and elevated blood glucose levels (15). Insulin resistance-induced hyperglycemia can result in abnormal regulation of immune cells, causing excessive production of pro-inflammatory factors, which subsequently contributes to the onset and progression of osteoporosis (16). Nonetheless, there remains a research gap regarding the potential of IR to predict adverse outcomes such as all-cause mortality and cardiovascular death events in patients with osteoporosis.

The hyperinsulinemic-euglycemic clamp (HECT) is widely regarded as the gold standard for evaluating IR (17). Another commonly employed method for assessing IR is the homeostasis model assessment of insulin resistance (HOMA-IR) (18). However, due to the complexity and cost associated with these two methodologies, neither is suitable for routine clinical practice. The triglycerides and glucose index (TyG), triglycerides to high-density lipoprotein cholesterol ratio (TG/HDL-C), and METS-IR are recognized as effective biomarkers for the identification of IR, given their significant association with HECT (19–21). Additionally, there is an elevated risk of osteoporosis linked to excessive fat mass resulting from obesity and the accumulation of visceral fat (22). Recent research indicates that combining the TyG index with body mass index (TyG-BMI) significantly enhances its effectiveness in evaluating IR (23). Furthermore, another study suggests that TyG-BMI can serve as a predictor of one-year all-cause mortality in patients with heart failure (24). Therefore, employing the TyG-BMI index as a metric for assessing insulin resistance to predict all-cause and cardiovascular mortality risk in patients with osteoporosis emerges as a practical and feasible approach. This study aims to provide clinicians with more convenient predictive tools and reduce the mortality risk for affected patients.

## Methods

### Study design and data collection

This research constitutes a prospective study focusing on patients diagnosed with osteoporosis who were admitted to our hospital between January 1, 2018, and December 31, 2020, with follow-up concluding on December 31, 2024. This study adheres to the ethical principles outlined in the Declaration of Helsinki (1964) and its subsequent amendments and has received approval from the institutional ethics committee. Clinical data only is collected, ensuring the exclusion of personal or identifying information. The study obtained ethical committee approval and a waiver for informed consent.

### Patient selection

The cohort of this study consists of adult participants diagnosed with osteoporosis. The exclusion criteria are as follows: (1) individuals under the age of 18; (2) participants with incomplete data on bone mineral density (BMD), fasting blood glucose (FBG), triglycerides (TG), and body mass index (BMI); (3) individuals lacking data on relevant covariates; and (4) participants lost to follow-up or who declined participation in the study for any reason.

### The diagnosis of OP and the measurement of indices such as TyG-BMI

Osteoporosis was diagnosed through bone mineral density (BMD) assessment using dual-energy X-ray absorptiometry (DXA). Measurements and recordings were conducted by radiology technicians with specialized training. Participants were classified into three groups based on their BMD at the total femur (TF), femoral neck (FN), or lumbar spine (LS): normal, osteopenia, or osteoporosis. Osteoporosis was distinguished according to the criteria established by Looker et al. (25). Mean BMD values in young adults aged 18 to 25 years were used as reference values for the general male and female population. Individuals with a BMD score more than 2.5 standard deviations below the reference value were diagnosed with osteoporosis, while those with a BMD score more than 1.0 standard deviation above the reference value were considered to have a normal BMD. Participants with BMD values between these thresholds were classified as having osteopenia.

Venous blood glucose levels and fasting triglyceride levels of patients diagnosed with osteoporosis were collected within 24 hours of admission, and the TyG-BMI index was subsequently calculated. The TyG-BMI index was derived using the following formulas: BMI = weight (kg)/height (m²); TyG index = Ln[1/2 × fasting glucose (mg/dL) × fasting triglycerides (mg/dL)]; TyG-BMI = TyG index × BMI. The METS-IR was calculated based on fasting blood glucose, fasting triglycerides, high-density lipoprotein cholesterol, and BMI. The formula used for this calculation is as follows: METS-IR = Ln((2 × FBG (mg/dL)) + TG (mg/dL)) × BMI (kg/m²)/Ln(HDL-C (mg/dL)). Additionally, the formula for calculating the TG/HDL-C ratio is: TG/HDL-C = TG (mg/dL) ÷ HDL-C (mg/dL).

### Ascertainment of mortality

In the cohort study, patient mortality was assessed through subsequent follow-up, with the follow-up period extending from January 1, 2021, to December 31, 2024. The follow-up results were documented by two qualified medical personnel. The mortality outcomes included all-cause mortality and cardiovascular mortality, with patients who died from other causes being censored at the time of death. Furthermore, the classification of causes of death adhered to the International Classification of Diseases, 10th Revision (ICD-10) (26).

### Variables

Data were collected by extracting information from the hospital’s health information system. The data were retrieved and organized by three trained researchers (YHL, XWX, and YZL) from electronic medical records. The records included the following: (1) demographic information (age, gender, smoking and drinking status); (2) comorbidities (hypertension, diabetes); (3) laboratory-related indicators (BMI, blood urea nitrogen, creatinine, uric acid, total bilirubin, fasting blood glucose levels, fasting triglyceride levels, calcium concentration, phosphorus concentration, high-density lipoprotein cholesterol, low-density lipoprotein cholesterol). To ensure the impartiality of data extraction, all data underwent secondary review by senior researchers (WBD and ZQG).

### Statistical analysis

Continuous variables were presented as weighted means with standard errors (± SE), while categorical variables were reported as frequencies and weighted proportions. To evaluate the differences across the TyG-BMI quartiles (Q1–Q4), the Chi-square test was utilized for categorical variables, and the Kruskal–Wallis test was employed for continuous variables. Additionally, a multivariate Cox proportional hazards regression analysis was conducted to examine the relationship between TyG-BMI and mortality in patients with osteoporosis. Model 2 incorporated adjustments for demographic characteristics, whereas Model 3 accounted for all covariates. The results were presented as hazard ratios (HRs) with 95% confidence intervals (CIs). Furthermore, the proportional hazards assumption underlying the Cox model was rigorously tested.

To compare all-cause mortality and cardiovascular mortality across different quartiles (Q1-Q4) for four indicators (TyG-BMI, TyG, METS-IR, TG/HDL-C), the mortality rates for each quartile will be presented in bar charts. A chi-square test will be employed to assess differences in mortality between the quartiles, with a p-value < 0.05 indicating statistical significance. All statistical analyses were performed using IBM SPSS Statistics version 25.0 and R version 4.3.1.

Additionally, receiver operating characteristic (ROC) curves were constructed for TyG-BMI, TyG, METS-IR, and TG/HDL-C in relation to all-cause mortality and cardiovascular mortality. These curves were utilized to evaluate the differential predictive capabilities of these four indices regarding mortality in patients with osteoporosis. A Delong test was concurrently performed for validation, employing the DeLong method to calculate the differences between the two area under the curve (AUC) values and their standard errors. Based on the computed differences and standard errors, a z-test was used to assess the significance of these differences. Furthermore, the restricted cubic spline method was applied to visualize variations in survival risk across different levels of the TyG-BMI index.

Subgroup analyses were conducted to evaluate the potential impact of additional variables on the relationship between the TyG-BMI index and mortality, thereby validating the robustness of our findings and identifying populations at a survival disadvantage. All p-values were two-sided, with statistical significance defined as P < 0.05. It should be noted that subgroup analyses involving multiple comparisons were conducted as exploratory analyses to generate hypotheses, and the relevant results should be interpreted with caution. Statistical analyses were performed using IBM SPSS Statistics version 25.0 and R version 4.3.1.

## Results

**Figure 1:**
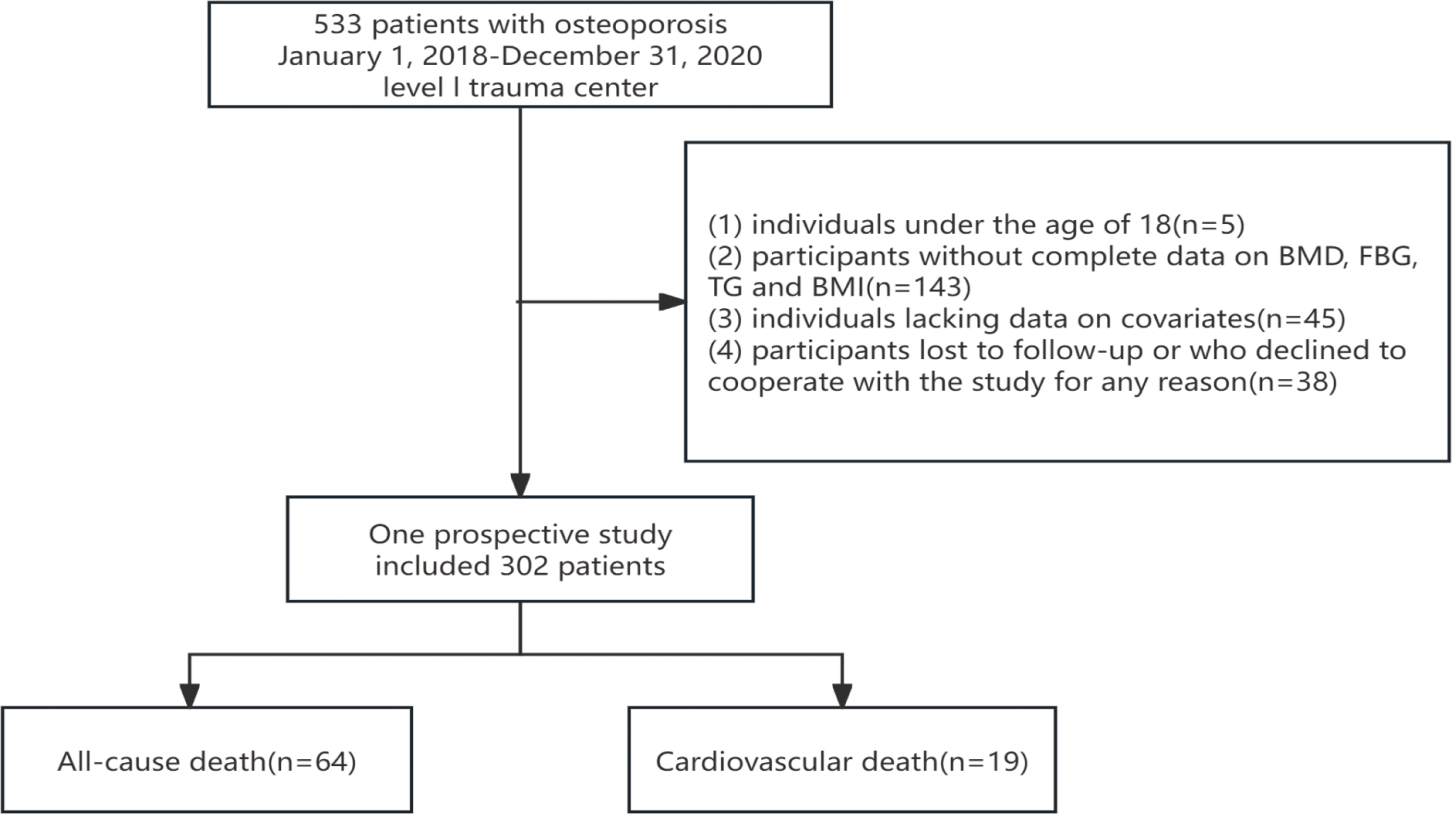
Selection process for study cohorts.

This study included a total of 302 patients who met the diagnostic criteria for osteoporosis. During the subsequent follow-up period, 64 cases of all-cause mortality and 19 cases of cardiovascular-related mortality were recorded among individuals diagnosed with osteoporosis (see **Figure 1**). The average age of the participants was 69.3 ± 12.4 years, with 72.2% being female and 27.8% male. **Table 1** provides a comprehensive overview of the baseline characteristics of the patients stratified by quartiles of the TyG-BMI index, revealing significant inter-group differences in factors such as diabetes prevalence, BMI, and blood urea nitrogen (p < 0.05).

**Table 1:** Baseline characteristics of the study cohort.

Study variables	Total (n=302)	Quartiles of TyG-BMI index				P-value
		Q1 ≤ 198.94 (n=76)	198.94<Q2 ≤ 220.43 (n=75)	220.43<Q3 ≤ 239.82 (n=75)	Q4>239.82 (n=76)	
Age,years	69.3 ± 12.4	68.37 ± 16.27	69.24 ± 11.70	69.15 ± 10.69	70.57 ± 10.26	0.75
Sex,n(%)						0.31
Female	218 (72.2%)	54 (71.05%)	55 (73.33%)	49 (65.33%)	60 (78.95%)	
Male	84 (27.8%)	22 (28.95%)	20 (26.67%)	26 (34.67%)	16 (21.05%)	
Smoking,n(%)						0.88
Yes	18 (6.0%)	5 (6.58%)	3 (4.00%)	5 (6.67%)	5 (6.58%)	
No	284 (94.0%)	71 (93.42%)	72 (96.00%)	70 (93.33%)	71 (93.42%)	
Alcohol use,n(%)						0.68
Yes	16 (5.3%)	5 (6.58%)	4 (5.33%)	2 (2.67%)	5 (6.58%)	
No	286 (94.7%)	71 (93.42%)	71 (94.67%)	73 (97.33%)	71 (93.42%)	
Diabetes,n(%)						**<0.01**
Yes	64 (21.2%)	7 (9.21%)	7 (9.33%)	14 (18.67%)	36 (47.37%)	
No	238 (78.8%)	69 (90.79%)	68 (90.67%)	61 (81.33%)	40 (52.63%)	
Hypertension,n(%)						0.40
Yes	109 (36.1%)	22 (28.95%)	26 (34.67%)	30 (40.00%)	31 (40.79%)	
No	193 (63.9%)	54 (71.05%)	49 (65.33%)	45 (60.00%)	45 (59.21%)	
BMI kg/m2	25.5 ± 3.1	21.92 ± 1.46	24.46 ± 1.50	26.45 ± 1.17	29.19 ± 1.99	**<0.01**
Fasting glucose (mg/dL)	106.3 ± 37.1	91.87 ± 19.06	104.88 ± 36.55	98.38 ± 23.20	129.79 ± 49.62	**<0.01**
Fasting triglyceride (mg/dL)	128.5 ± 66.1	103.80 ± 40.16	118.71 ± 46.05	136.63 ± 68.12	154.71 ± 88.31	**<0.01**
HDL(mmol/l)	1.2 ± 0.3	1.23 ± 0.34	1.26 ± 0.28	1.17 ± 0.29	1.16 ± 0.31	0.14
LDL(mmol/l)	3.0 ± 0.9	2.91 ± 0.79	3.15 ± 0.87	3.09 ± 1.15	2.88 ± 0.92	0.23
Total calcium(mmol/l)	2.9 ± 12.6	15.33 ± 8.11	15.28 ± 5.63	14.32 ± 7.38	18.21 ± 17.48	0.14
Phosphorus (mmol/l)	1.2 ± 0.2	1.19 ± 0.24	1.16 ± 0.18	1.18 ± 0.25	1.15 ± 0.23	0.80
Uric acid (μmol/L)	303.6 ± 95.4	300.25 ± 104.03	287.07 ± 81.79	327.33 ± 93.96	299.99 ± 97.78	0.07
STB(μmol/L)	15.8 ± 10.8	15.33 ± 8.11	15.28 ± 5.63	14.32 ± 7.38	18.21 ± 17.48	0.14
BUN(mmol/l)	6.4 ± 2.2	6.27 ± 2.07	6.10 ± 1.89	7.00 ± 2.50	6.18 ± 2.16	**0.04**
Cr(μmol/L)	63.0 ± 20.4	61.38 ± 16.23	61.39 ± 18.20	67.99 ± 27.21	61.32 ± 17.58	0.11

BUN, blood urea nitrogen; Cr, creatinine; LDL, Low-Density Lipoprotein Cholesterol; HDL, High-Density Lipoprotein Cholesterol; STB, Total bilirubin. P-values of <0.05 were shown in bold.

**Figure 2:**
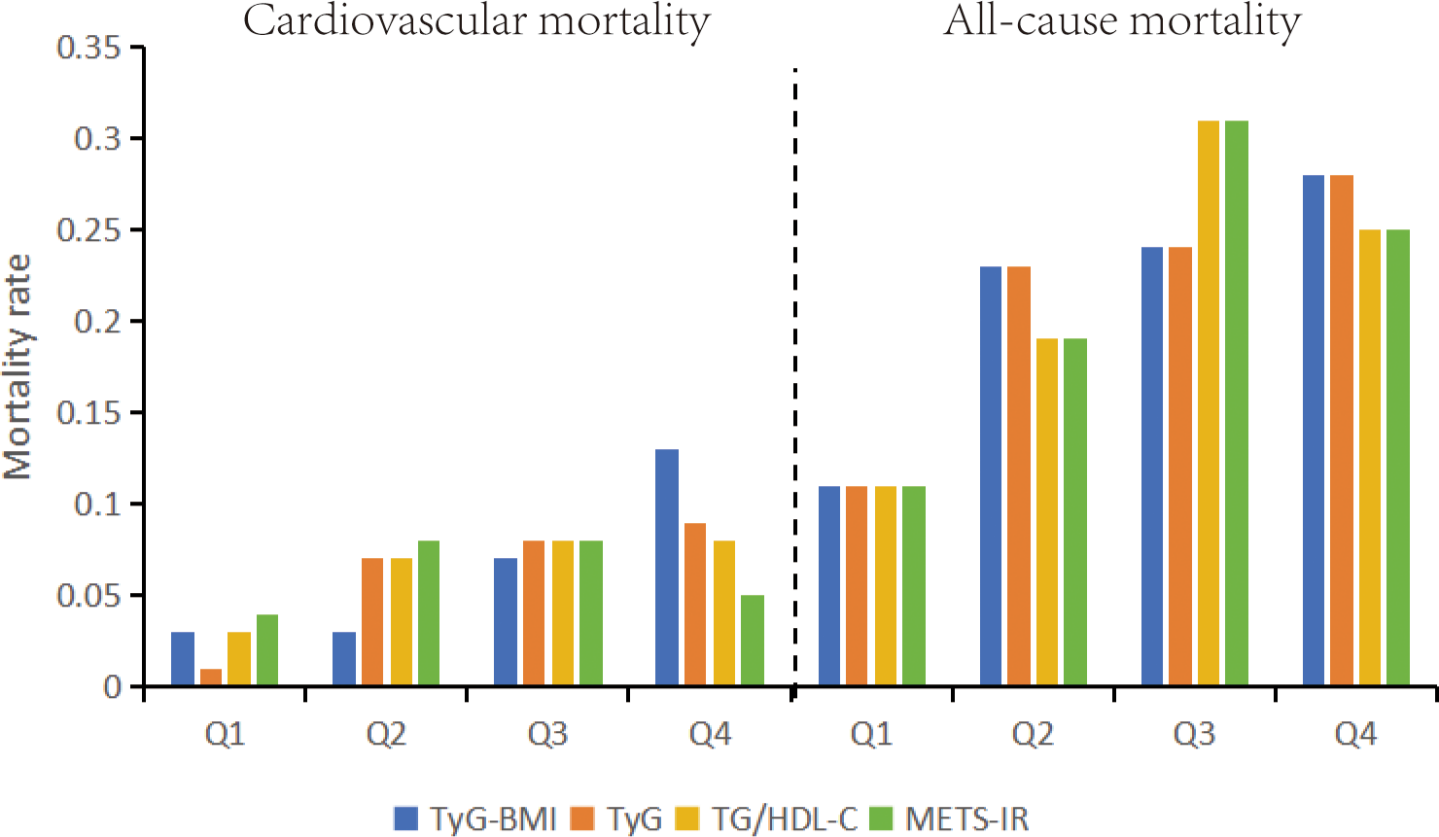
All-cause mortality and cardiovascular mortality bar graphs based on quartiles of TyG-BMI index, TyG index, METS-IR, TG/HDL-C stratification. TyG-BMI, Triglyceride Glucose-body Mass; TyG, Triglyceride and Glucose; METS-IR, metabolic score for Insulin resistance; TG/HDL-C, triglyceride/high-density lipoprotein cholesterol.

**Figure 2** presents a bar chart depicting the relationship between the quartiles of four indices and mortality rates. It is apparent that both all-cause mortality and cardiovascular mortality exhibit a gradual increase with the elevation of the TyG-BMI and TyG indices. The TyG-BMI index shows all-cause mortality rates of 11%, 23%, 24%, and 28% (P < 0.01) and cardiovascular mortality rates of 3%, 3%, 7%, and 13% (P < 0.01). Similarly, the TyG index demonstrates all-cause mortality rates of 11%, 23%, 24%, and 28% (P < 0.01) and cardiovascular mortality rates of 1%, 7%, 8%, and 9% (P < 0.01).

**Figure 3:**
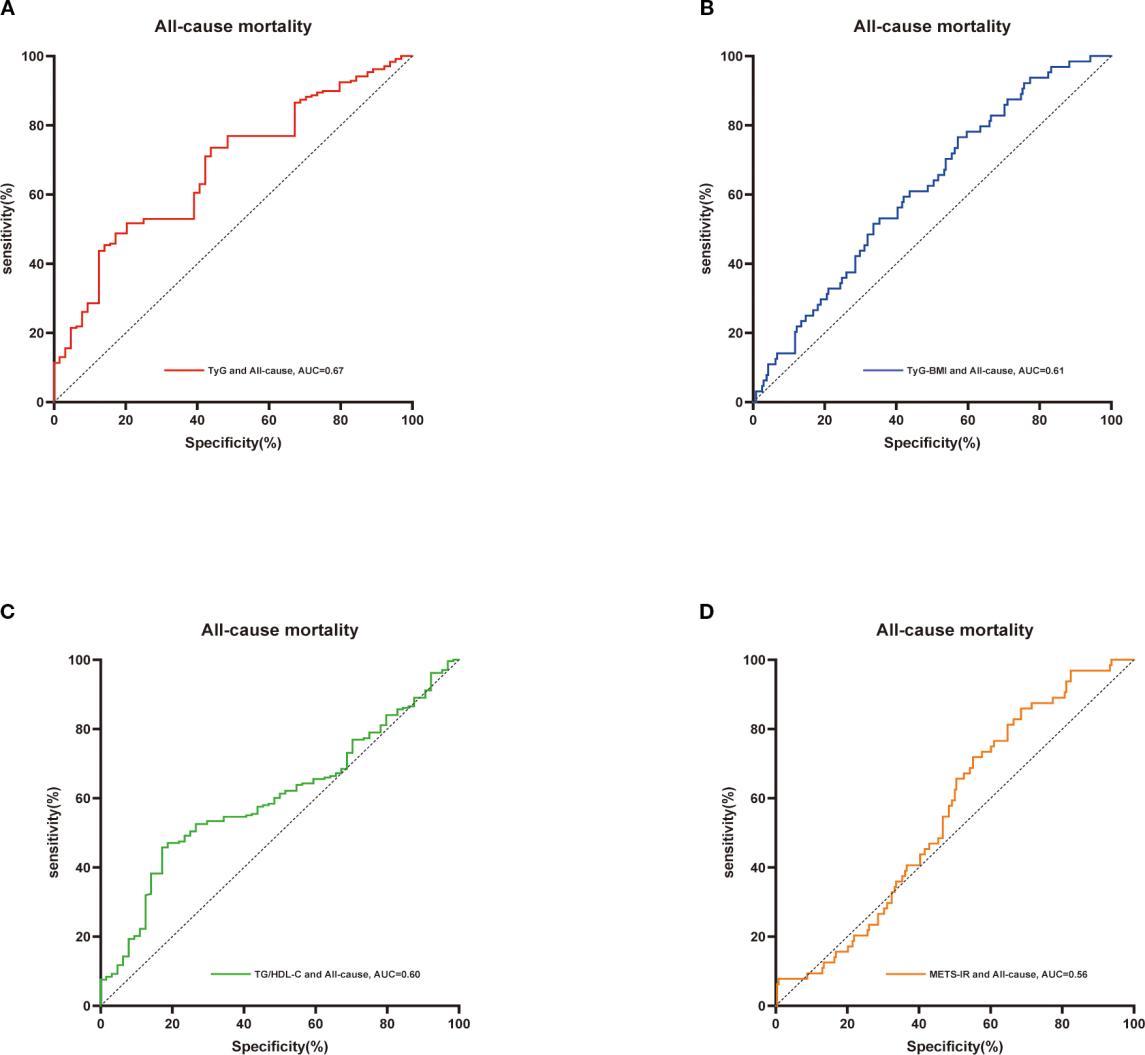
ROC curves were utilized to assess the differences in predictive abilities of TyG **(A)**,TyG-BMI index **(B)**, TG/HDL-C **(C)** and METS-IR **(D)** for all-cause mortality in osteoporosis patients. ROC, receiver operating characteristic curve; AUC, area under the curve.

**Figure 4:**
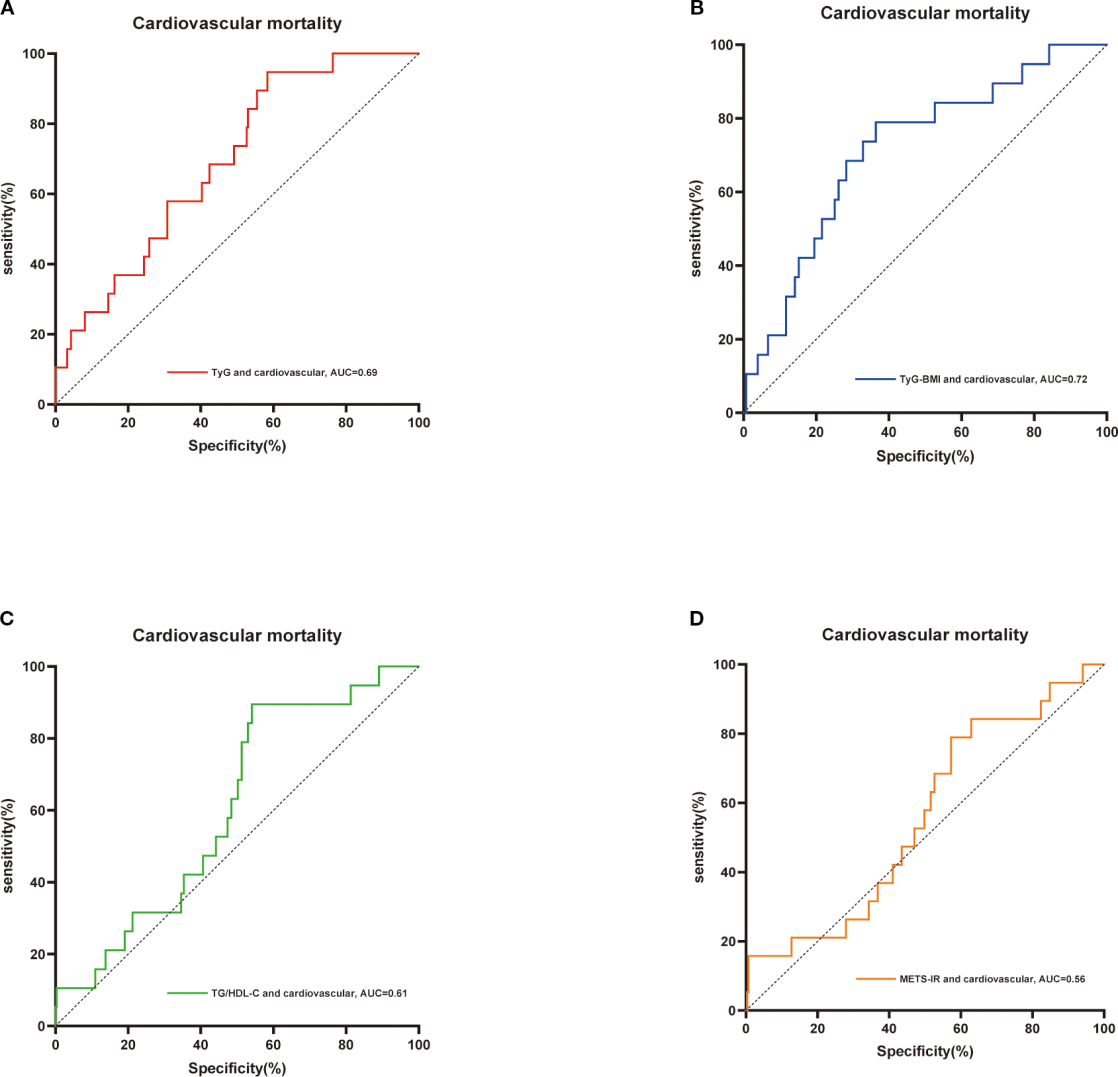
ROC curves were utilized to assess the differences in predictive abilities of TyG index **(A)**,TyG-BMI index **(B)**, TG/HDL-C index **(C)** and METS-IR index **(D)** for cardiovascular mortality in osteoporosis patients. ROC, receiver operating characteristic curve; AUC, area under the curve.

Additionally, we employed ROC curves and the DeLong test to assess the differences in the predictive capabilities of the TyG-BMI, TyG, METS-IR, and TG/HDL-C indices for mortality in patients with osteoporosis. As illustrated in **Figures 3**, **4** and **Table 2**, the TyG-BMI index exhibited superior overall performance in predicting both all-cause mortality and cardiovascular mortality when compared to the other three indices, which aligns with previous research findings.

**Table 2:** The results of the Delong test relating TyG-BMI, TyG, METS-IR, and TG/HDL-C to all-cause mortality and cardiovascular mortality in patients with osteoporosis.

Predictive indicator	TyG-BMI	TyG	METS-IR	TG/HDL-C
Allcause				
AUC	0.61	0.67	0.56	0.60
DeLong pvalue	reference	0.16	0.19	0.76
Bootstrap 95% CI	0.54-0.69	0.60-0.74	0.49-0.63	0.52-0.67
Cardiovascular				
AUC	0.72	0.69	0.56	0.61
DeLong p value	reference	0.78	0.02	0.16
Bootstrap 95% CI	0.60-0.82	0.59-0.79	0.43-0.68	0.50-0.72

TyG-BMI, Triglyceride Glucose-body Mass; TyG, Triglyceride and Glucose; METS-IR, metabolic score for Insulin resistance; TG/HDL-C, triglyceride/high-density lipoprotein cholesterol.

The Cox regression analysis presented in **Table 3** outlines the nuanced association between the TyG-BMI index and mortality rates, considering both continuous variables and quartile groupings. When the TyG-BMI index is treated as a continuous variable in a simple linear model, the results reveal a statistically significant positive correlation between TyG-BMI levels and all-cause mortality. This relationship remains robust even after adjusting for various covariates (Models 2 and 3). Specifically, for each one-unit increase in TyG-BMI levels, the risk of all-cause mortality rises by 1% (95% CI: 1.00-1.02). When the TyG-BMI index is categorized into quartiles, individuals in the second, third, and fourth quartiles (Q2, Q3, Q4) exhibit a progressively higher risk of all-cause mortality compared to those in the lowest quartile (Q1) of individuals with osteoporosis (Q2: HR: 2.57, 95% CI: 1.09-6.08; Q3: HR: 2.72, 95% CI: 1.14-6.49; Q4: HR: 2.79, 95% CI: 1.16-6.73). Furthermore, a significant positive correlation is observed between TyG-BMI levels and cardiovascular mortality. For each one-unit increase in TyG-BMI levels, the risk of cardiovascular mortality rises by 2% (95% CI: 1.01-1.04). In contrast to all-cause mortality, when the TyG-BMI index is classified into quartiles, only individuals in the fourth quartile (Q4) (CI: 1.19-33.80) demonstrate a statistically significant increase in cardiovascular mortality risk compared to those in the lowest quartile (Q1).

**Table 3:** Associations between the TyG-BMI index and all-cause and cardiovascular mortality in patients with osteoporosis.

Group	Model1		Model2		Model3	
	HR (95% CI)	p value	HR (95% CI)	p value	HR (95% CI)	p value
All-cause mortality						
TyG-BMI	1.01(1.00-1.02)	**<0.01**	1.01(1.01-1.02)	**0.01**	1.01(1.00-1.02)	**0.01**
Q1	Reference		Reference		Reference	
Q2	2.41(1.04-5.60)	**0.04**	2.44(1.05-5.68)	**0.04**	2.57(1.09-6.08)	**0.03**
Q3	2.85(1.23-6.59)	**0.01**	2.87(1.24-6.62)	**0.01**	2.72(1.14-6.49)	**0.02**
Q4	3.29(1.45-7.44)	**<0.01**	3.25(1.43-7.40)	**0.01**	2.79(1.16-6.73)	**0.03**
Cardiovascular mortality						
TyG-BMI	1.02(1.01-1.03)	**<0.01**	1.02(1.01-1.04)	**<0.01**	1.02(1.01-1.04)	**<0.01**
Q1	Reference		Reference		Reference	
Q2	1.13(0.16-8.06)	0.90	1.17(0.16-8.33)	0.88	1.47(0.19-11.63)	0.71
Q3	3.19(0.61-16.58)	0.17	3.28(0.63-17.14)	0.16	4.15(0.69-25.15)	0.12
Q4	6.32(1.38-28.93)	**0.02**	6.59(1.42-30.69)	**0.02**	6.33(1.19-33.80)	**0.03**

Model 1, unadjusted. Model 2, adjusted for gender and age. Model 3, adjusted for all covariates including gender, age, hypertension, diabetes, alcohol consumption, and smoking. P-values of <0.05 were shown in bold.

**Figure 5:**
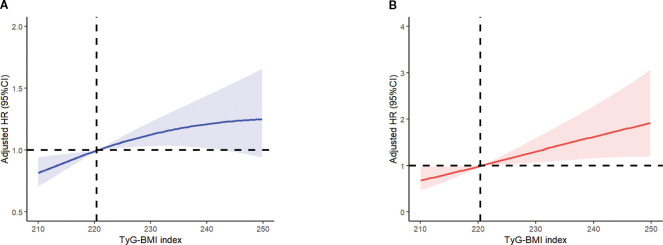
Multivariable adjusted spline curves for associations of the TyG-BMI index with all-cause **(A)** and cardiovascular mortality **(B)** in respondents with osteoporosis. All covariates have been adjusted. The solid line and red area represent the estimated values and their corresponding 95% CI. CI, confidence interval; TyG-BMI index, Triglyceride Glucose-body Mass.

The restricted cubic spline plot clearly illustrates the linear relationship between TyG-BMI and both all-cause mortality and cardiovascular mortality, with these results remaining consistent after adjusting for all covariates (see **Figure 5**). As the findings suggest, the risk of both all-cause mortality and cardiovascular mortality increases progressively with the rising levels of the TyG-BMI index, which aligns with our previous findings.

**Figure 6:**
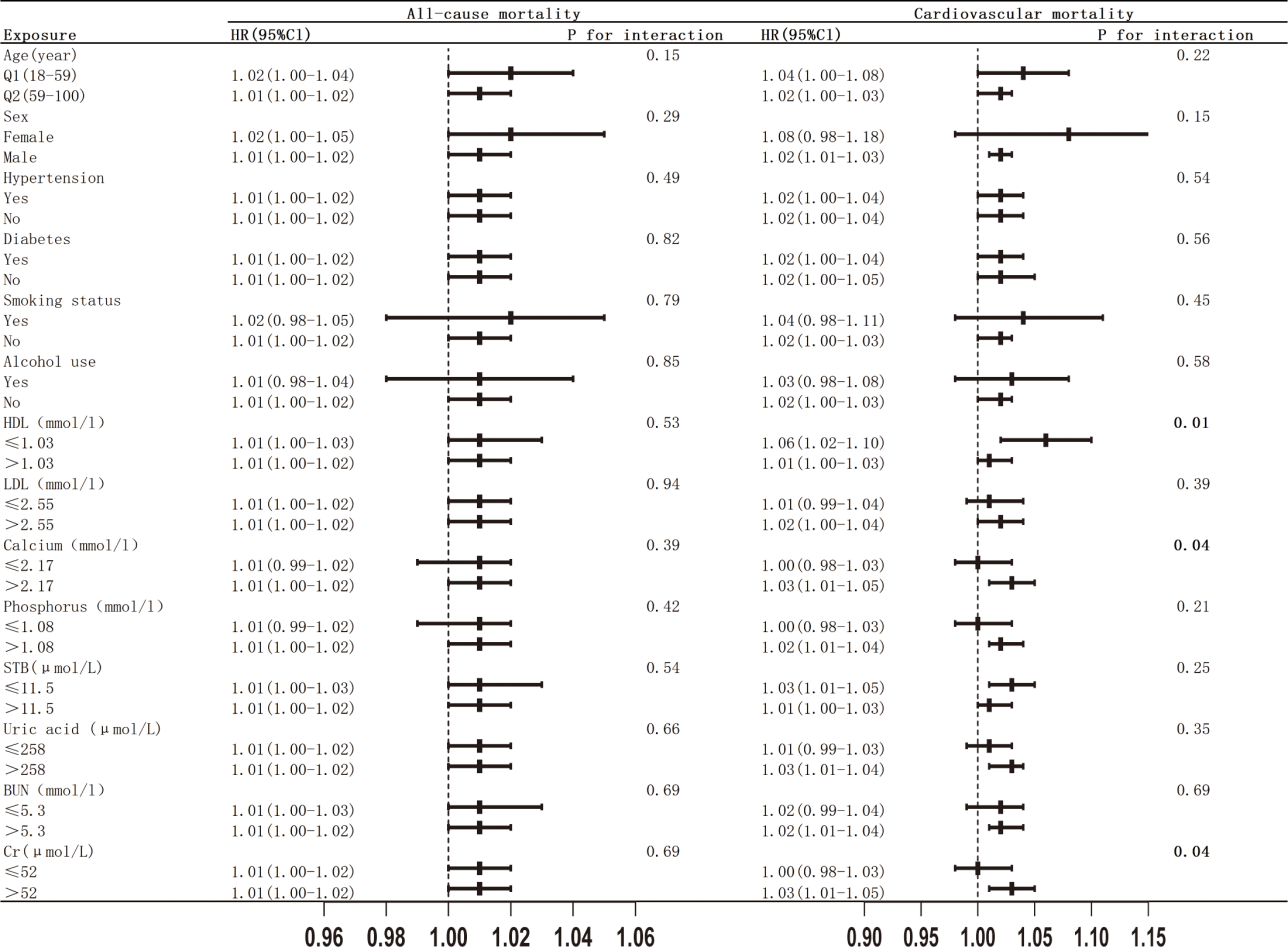
Subgroup analysis of the associations between TyG-BMI index with all-cause and cardiovascular mortality.

To further enhance our understanding of the relationship between a high TyG-BMI index at admission and various other variables, we conducted a comprehensive subgroup analysis (**Figure 6**). The results reveal that for all-cause mortality, no significant interactions were observed between high TyG-BMI index levels and any of the covariates examined (all interaction p-values > 0.05). The lack of significant interactions suggests that the effect of a high TyG-BMI index on all-cause mortality in patients with osteoporosis remains relatively consistent, irrespective of factors such as age, comorbidities, or other laboratory indicators. In contrast, for cardiovascular mortality, potential effect modifications were suggested by HDL levels (interaction p-value = 0.01), calcium ion levels (interaction p-value = 0.04), and creatinine levels (interaction p-value = 0.04). These exploratory findings suggest that clinicians might pay attention to the risk of cardiovascular mortality in osteoporotic patients with low HDL levels, elevated calcium ion concentrations, and high creatinine levels. However, it is important to note that these interaction analyses involved multiple comparisons, which increases the possibility of false-positive findings. Therefore, these results should be interpreted as exploratory and hypothesis-generating, and they require confirmation in future research.

## Discussion

In this prospective study, we systematically investigated, for the first time, the relationship between the TyG-BMI index and both all-cause mortality and cardiovascular mortality in patients with osteoporosis. Our findings revealed that, regardless of whether the TyG-BMI index is treated as a continuous variable or analyzed through quartile classification, it exhibits a significant positive correlation with all-cause mortality in osteoporotic patients. In the fourth quartile of the TyG-BMI index, patients with osteoporosis demonstrated a higher risk of cardiovascular mortality (HR: 6.33, 95% CI: 1.19-33.80). After adjusting for various potential confounders, including age, gender, hypertension, diabetes, smoking and drinking habits, and other laboratory indicators, this association remained highly significant. Overall, maintaining low levels of the TyG-BMI index may offer better long-term outcomes for patients with osteoporosis. Additionally, in comparisons of predictive capabilities for both all-cause mortality and cardiovascular mortality with other indices, we found that the TyG-BMI index exhibited the most robust predictive capacity. Therefore, the TyG-BMI index may hold significant preventive value in assessing the severity and clinical outcomes of patients with osteoporosis.

A substantial body of research has established an association between insulin receptors (InsR) and bone mineral density (BMD); however, the findings remain inconsistent. For instance, Mihai G et al. identified an inverse relationship between markers of insulin resistance and femoral neck BMD in postmenopausal women (27). In contrast, a study conducted by Dennison EM et al. reported that total femoral density and femoral neck bone density exhibited a positive correlation with insulin resistance measurements, particularly among females (28). Furthermore, another investigation revealed that HOMA-β was negatively correlated with low levels of HOMA-IR in individuals with osteoporosis (HOMA-IR < 2). However, this association was not significant when HOMA-IR was greater than or equal to 2 (29). These discrepancies in findings may be attributed to variations in study populations and methodologies employed for assessing insulin resistance.

Insulin initiates a cascade of downstream signaling pathways, notably the PI3K/Akt pathway, through the activation of the insulin receptor (InsR). The insulin signaling pathway plays a crucial role in regulating osteoblast-mediated bone formation and osteoclast-mediated bone resorption (30, 31). Both osteoblasts and osteoclasts possess receptors for insulin and insulin-like growth factor-1 (IGF-1). Deficiencies or dysfunctions in these receptors can result in systemic insulin resistance (IR) and site-specific insulin resistance in bone tissue, leading to a decrease in both the quantity and functionality of osteoblasts (31). This decline consequently activates osteoclasts and promotes bone resorption, ultimately contributing to the development of osteoporosis (32). Moreover, insulin resistance is frequently associated with chronic low-grade inflammation, wherein inflammatory factors inhibit osteoblast function while enhancing osteoclast activity, resulting in diminished bone density (16, 33). Furthermore, the occurrence of oxidative stress, coupled with inflammatory processes and dysregulation of microRNAs (miRNAs) that modulate gene expression at the post-transcriptional level, exacerbates osteoclastogenesis and impairs osteoblastogenesis. This is mediated through mechanisms involving MAPK activation and transcription factor modulation, culminating in an increased risk of osteoporosis (34).

The TyG index, a biological parameter calculated as the product of triglycerides (TG) and fasting plasma glucose (FPG), has been established as an effective alternative marker for assessing insulin resistance (IR) (19). The TyG-BMI index represents the combined effects of the TyG index and body mass index (BMI). Research has demonstrated that excessive fat accumulation due to obesity increases the risk of osteoporosis (22). Obese individuals release leptin from adipocytes, which, through a central regulatory mechanism involving the hypothalamus, inhibits bone formation, thereby contributing to osteoporosis (35). Furthermore, obesity is linked to elevated levels of inflammatory mediators, including C-reactive protein (CRP), tumor necrosis factor-α (TNF-α), and interleukin-6 (IL-6). Both TNF-α and IL-6 promote osteoclast-mediated bone resorption (36, 37). Recent studies have indicated that combining the TyG index with BMI (TyG-BMI) significantly enhances its utility in assessing insulin resistance (23).

It is important to emphasize that the TyG-BMI index, while serving as a surrogate marker of insulin resistance and metabolic health, provides only an indirect assessment of bone status. However, this index does not account for key determinants of bone fragility, such as bone geometry, microarchitecture, and material properties (38). These factors are critical components of bone strength and fracture risk, operating independently of BMD (39). Consequently, the observed association may primarily reflect the adverse biological effects of metabolic dysregulation on bone remodeling rather than a direct measurement of mechanical competence. Given these limitations, future studies incorporating advanced imaging techniques—such as high-resolution peripheral quantitative computed tomography (HR-pQCT) or magnetic resonance imaging (MRI)—are warranted (40). Such methodologies could provide a deeper understanding of the specific impacts of insulin resistance on bone quality beyond mere density.

IR is strongly associated with cardiovascular diseases (41). Our research indicates that elevated levels of the TyG-BMI index correlate with an increased rate of cardiovascular mortality in patients with osteoporosis. This finding is consistent with prior studies on the TyG index and cardiovascular mortality rates (42, 43). The underlying mechanism may involve the widespread presence of chronic low-grade inflammation in osteoporosis patients, which significantly heightens the risk of irreversible cardiovascular events. This is mediated through shared pathological mechanisms, such as immune-inflammatory responses, oxidative stress, and endothelial dysfunction (11, 44, 45).

It is noteworthy that this study found a significant positive linear correlation between the TyG-BMI index and cardiovascular mortality in patients with osteoporosis. This finding contrasts with a recent study reporting a U-shaped nonlinear relationship in patients with osteoarthritis (OA) (46). We propose that this discrepancy may stem from the distinctly different pathophysiological mechanisms underlying these two conditions, despite their close association with IR. Firstly, the nature of the diseases and the core role of IR differ. In osteoporosis, the detrimental effects of IR on bone are primarily realized through the inhibition of osteoblast function and the promotion of osteoclast activity, a process more clearly associated with chronic inflammation and oxidative stress in a linear dose-response manner (11, 30, 31, 44, 45). Therefore, the degree of IR is more likely to exhibit a monotonic increasing relationship with the risk of cardiovascular mortality. In contrast, the role of IR in osteoarthritis is more complex. In addition to similar inflammatory and metabolic harms, insulin itself exerts crucial direct protective effects on articular cartilage, such as enhancing the synthesis of type II collagen and proteoglycans, and inhibiting the expression of collagenase and matrix metalloproteinases (MMP)-1 and 13 (47). Consequently, extremely low insulin levels may lead to the absence of this protective signaling, resulting in an increased risk at the left end of the U-shaped curve. In other words, for OA joints, there exists a tipping point where both excessively high and low insulin levels are detrimental; however, for osteoporotic bones, the harmful effects of IR may be more unidirectional. Secondly, differences in populations and comorbidities may also contribute to the varying forms of this association. OA patients often exhibit higher obesity rates and differing fat distribution patterns, which may influence the biological significance represented by the BMI component of the TyG-BMI index (48). Additionally, variations in commonly used medications, the extent of activity limitation, and lifestyle differences between the two patient groups may act as confounding factors, modifying the ultimate association between TyG-BMI and mortality.

Recent studies have established that both hypoglycemia and hyperglycemia are associated with an increased risk of falls in hospitalized populations (49). This relationship stems from dual mechanisms whereby high and low blood glucose levels elevate risk through chronic complications and acute neurological dysfunction, necessitating meticulous regulation to maintain optimal glycemic control (50–52). Moreover, the TyG-BMI index serves as a comprehensive indicator of insulin resistance and obesity. An increase in this index not only reflects the state of insulin resistance but also continuously indicates the severity of metabolic dysregulation. It contributes to adverse events through mechanisms such as immune-inflammation responses, oxidative stress, and endothelial dysfunction (44, 45). Consequently, the TyG-BMI index exhibits a linear growth pattern, suggesting that clinical interventions should actively aim to reduce this index to achieve sustained improvements in cardiovascular disease outcomes.

In this study, although our analysis has controlled for several known confounding variables, we recognize that there are still some unmeasured factors that may influence the results, particularly in the elderly population with osteoporosis. Specifically, factors such as bone quality, medication use, and muscle mass could have significant impacts on our findings. For instance, a decline in bone quality may not only affect fracture risk but also directly influence mortality rates (53). Furthermore, certain medications used to treat osteoporosis, such as bisphosphonates or selective estrogen receptor modulators, may alter metabolic indices and their relationship with mortality (54). Additionally, muscle mass, as a crucial component of overall health, can affect functional capacity and nutritional status, subsequently impacting mortality risk (55). Therefore, future research should consider incorporating these key parameters to provide a more comprehensive understanding of the health status of individuals with osteoporosis.

Our research is based on a long-term follow-up of hospitalized patients with osteoporosis and aims to elucidate the relationship between the TyG-BMI index and both all-cause and cardiovascular mortality in this patient population. By identifying high-risk individuals, the TyG-BMI index can serve as a valuable tool for physicians in formulating personalized monitoring and treatment strategies, ultimately enhancing patient outcomes. However, additional studies are required to confirm the clinical relevance and practical applicability of the TyG-BMI index in evaluating osteoporosis-related risks.

## Limitations

This study has several notable limitations. Firstly, it is based on a prospective investigation conducted in a single-center setting, which may limit the diversity and representativeness of the sample. Consequently, the findings may not adequately reflect the circumstances encountered across different regions and ethnic backgrounds. Future research should include a more diverse population, encompassing different ethnicities, age groups, and individuals with a wider range of chronic diseases. By expanding the diversity of the sample, the external validity of the study findings can be enhanced, ultimately providing better guidance for public health practices. Secondly, during the prolonged follow-up after admission, variables such as fasting blood glucose, fasting triglyceride levels, and body mass index (BMI) may exhibit fluctuations. While we accounted for baseline values of these parameters recorded at the time of admission to mitigate confounding effects, we did not assess their relationships over time. Thirdly, the patients’ socioeconomic status, educational attainment, and their influence on health management may not have been sufficiently addressed, as these factors could significantly impact mortality rates and health outcomes. Fourth, although we observed several potential interactions in subgroup analyses for cardiovascular mortality, it is important to note that these analyses involved multiple comparisons. This increases the possibility that some of the observed associations may be due to chance. Therefore, these results should be interpreted as exploratory and hypothesis-generating rather than confirmatory. They are presented to guide future research and require validation in independent, large-scale prospective cohorts. Lastly, despite our thorough consideration of various covariates, unexplained confounding factors may still compromise the validity of our conclusions.

## Conclusion

Through the investigation of the characteristics of the TyG-BMI index as a marker of insulin resistance in patients with osteoporosis, we identified a linear relationship between TyG-BMI index levels in osteoporotic patients and both all-cause and cardiovascular mortality. Patients with osteoporosis exhibiting elevated TyG-BMI indices are at an increased risk of mortality. When compared to other composite indicators of insulin resistance, the TyG-BMI index demonstrates superior predictive capability. These findings offer clinicians a novel perspective and intervention targets for the timely identification and management of osteoporotic patients at heightened risk of mortality. Future studies should be conducted to further evaluate the role of this index in the prognostic management of individuals with osteoporosis.

## Data Availability

The raw data supporting the conclusions of this article will be made available by the authors, without undue reservation.
